# Underutilization of Influenza Antiviral Treatment Among Children and Adolescents at Higher Risk for Influenza-Associated Complications — United States, 2023–2024

**DOI:** 10.15585/mmwr.mm7345a2

**Published:** 2024-11-14

**Authors:** Aaron M. Frutos, Haris M. Ahmad, Dawud Ujamaa, Alissa C. O’Halloran, Janet A. Englund, Eileen J. Klein, Danielle M. Zerr, Melanie Crossland, Holly Staten, Julie A. Boom, Leila C. Sahni, Natasha B. Halasa, Laura S. Stewart, Olla Hamdan, Tess Stopczynski, William Schaffner, H. Keipp Talbot, Marian G. Michaels, John V. Williams, Melissa Sutton, M. Andraya Hendrick, Mary A. Staat, Elizabeth P. Schlaudecker, Brenda L. Tesini, Christina B. Felsen, Geoffrey A. Weinberg, Peter G. Szilagyi, Bridget J. Anderson, Jemma V. Rowlands, Murtada Khalifa, Marc Martinez, Rangaraj Selvarangan, Jennifer E. Schuster, Ruth Lynfield, Melissa McMahon, Sue Kim, Val Tellez Nunez, Patricia A. Ryan, Maya L. Monroe, Yun F. Wang, Kyle P. Openo, James Meek, Kimberly Yousey-Hindes, Nisha B. Alden, Isaac Armistead, Suchitra Rao, Shua J. Chai, Pam Daily Kirley, Ariana P. Toepfer, Fatimah S. Dawood, Heidi L. Moline, Timothy M. Uyeki, Sascha Ellington, Shikha Garg, Catherine H. Bozio, Samantha M. Olson

**Affiliations:** ^1^Influenza Division, National Center for Immunization and Respiratory Diseases, CDC; ^2^Epidemic Intelligence Service, CDC; ^3^General Dynamics Information Technology, Atlanta, Georgia; ^4^Seattle Children’s Research Institute, Seattle, Washington; ^5^Salt Lake County Health Department, Salt Lake City, Utah; ^6^Baylor College of Medicine, Houston, Texas; ^7^Texas Children’s Hospital, Houston, Texas; ^8^Vanderbilt University Medical Center, Nashville, Tennessee; ^9^University of Pittsburgh School of Medicine, Pittsburgh, Pennsylvania; ^10^UPMC Children’s Hospital of Pittsburgh, Pittsburgh, Pennsylvania; ^11^Public Health Division, Oregon Health Authority; ^12^University of Cincinnati College of Medicine, Cincinnati, Ohio; ^13^Cincinnati Children’s Hospital Medical Center, Cincinnati, Ohio; ^14^University of Rochester School of Medicine and Dentistry, Rochester, New York; ^15^UCLA Mattel Children’s Hospital, Los Angeles, California; ^16^New York State Department of Health; ^17^New Mexico Department of Health; ^18^University of Missouri-Kansas City School of Medicine, Kansas City, Missouri; ^19^Children’s Mercy Hospital, Kansas City, Missouri; ^20^Minnesota Department of Health; ^21^Michigan Department of Health & Human Services; ^22^Maryland Department of Health; ^23^Department of Pathology and Laboratory Medicine, Emory University School of Medicine, Grady Health System, Atlanta, Georgia; ^24^Division of Infectious Diseases, Emory University School of Medicine, Atlanta, Georgia; ^25^Georgia Emerging Infections Program, Georgia Department of Public Health; ^26^Research, Atlanta Veterans Affairs Medical Center, Decatur, Georgia; ^27^Connecticut Emerging Infections Program, Yale School of Public Health, New Haven, Connecticut; ^28^Colorado Department of Public Health and Environment; ^29^Department of Pediatrics, University of Colorado Anschutz Medical Campus, Aurora, Colorado, United States; ^30^California Emerging Infections Program, Oakland, California; ^31^Office of Readiness and Response, CDC; ^32^Coronavirus and Other Respiratory Viruses Division, National Center for Immunization and Respiratory Diseases, CDC.

**Summary**

**What is already known about this topic?**

Tens of thousands of children and adolescents are hospitalized each year in the United States with influenza. Both vaccination and antiviral treatment can reduce the risk for influenza complications.

**What is added by this report?**

Data from two national influenza surveillance networks indicate that antiviral treatment of hospitalized children and adolescents with influenza has declined from 70%– 86% during the 2017–18 season to <60% in 2023–24. Only 30% of children and adolescents at higher risk for influenza complications were prescribed antivirals during outpatient visits.

**What are the implications for public health practice?**

All hospitalized children and adolescents and those at higher risk for influenza complications seen in outpatient settings with suspected influenza should receive antivirals as soon as possible to reduce the risk for influenza complications.

## Abstract

Annually, tens of thousands of U.S. children and adolescents are hospitalized with seasonal influenza virus infection. Both influenza vaccination and early initiation of antiviral treatment can reduce complications of influenza. Using data from two U.S. influenza surveillance networks for children and adolescents aged <18 years with medically attended, laboratory-confirmed influenza for whom antiviral treatment is recommended, the percentage who received treatment was calculated. Trends in antiviral treatment of children and adolescents hospitalized with influenza from the 2017–18 to the 2023–2024 influenza seasons were also examined. Since 2017–18, when 70%–86% of hospitalized children and adolescents with influenza received antiviral treatment, the proportion receiving treatment notably declined. Among children and adolescents with influenza during the 2023–24 season, 52%–59% of those hospitalized received antiviral treatment. During the 2023–24 season, 31% of those at higher risk for influenza complications seen in the outpatient setting in one network were prescribed antiviral treatment. These findings demonstrate that influenza antiviral treatment is underutilized among children and adolescents who could benefit from treatment. All hospitalized children and adolescents, and those at higher risk for influenza complications in the outpatient setting, should receive antiviral treatment as soon as possible for suspected or confirmed influenza.

## Introduction

Annually, seasonal influenza virus infections among children and adolescents in the United States are estimated to result in millions of medical visits, tens of thousands of hospitalizations, and hundreds of deaths.^†^ Influenza hospitalization rates among children and adolescents are highest among those aged <1 year, and rates decrease with increasing age.^§^ Influenza vaccination and early initiation of antiviral treatment can reduce the risk for influenza complications (*1*,*2*). Prompt antiviral treatment has also been associated with lower odds of intensive care unit (ICU) admission and death among hospitalized children and adolescents with influenza (*3*). Antiviral treatment is recommended as soon as possible, and treatment of any person with suspected or confirmed influenza who is hospitalized; has severe, complicated, or progressive illness; or is at higher risk for influenza complications should not await laboratory confirmation (*4*,*5*). In addition to persons with certain underlying medical conditions, children aged <5 years are considered to be at higher risk for influenza complications; the highest risk is among those aged <2 years (*5*).

During the 2022–23 influenza season, underutilization of antiviral treatment was observed among hospitalized children and adolescents with laboratory-confirmed influenza compared with its use during seasons before the COVID-19 pandemic (*6*). This report examines antiviral treatment patterns among children and adolescents with laboratory-confirmed influenza who were hospitalized and among those at higher risk for influenza complications within the outpatient setting during the 2023–24 influenza season.

## Methods

### Data Collection

Data were collected from two U.S. influenza surveillance networks,^¶^ the Influenza Hospitalization Surveillance Network (FluSurv-NET) and the New Vaccine Surveillance Network (NVSN). For this analysis, patients were included from both networks during October 1, 2023–April 30, 2024. FluSurv-NET is an active, population-based influenza hospitalization surveillance network that collects data on persons of all ages. A FluSurv-NET case was defined as a hospitalization of a person of any age residing in the surveillance catchment area with laboratory-confirmed influenza from a clinically ordered test.** Data were collected through review of medical records using a standardized case report form on an age-, site-, and month of admission–stratified random sample of cases from 12 sites.^††^ All sampled children and adolescents aged <18 years from FluSurv-NET were included in the analyses. Cases that were not sampled, cases missing influenza antiviral treatment data, cases of nosocomial influenza, and cases among pregnant persons were excluded.

NVSN is an active, population-based surveillance network that collects data from children and adolescents aged <18 years with acute respiratory illness (ARI) in outpatient (outpatient clinics, urgent care clinics, and emergency departments) and hospital settings at seven sites.^§§^ An NVSN case was defined as ARI^¶¶^ and laboratory-confirmed influenza*** from a clinically ordered test in a child or adolescent aged <18 years living in the catchment area. For NVSN, all hospitalized patients with laboratory-confirmed influenza were included, but in the outpatient setting, cases were only included if the patients were recommended to receive influenza antiviral treatment based on CDC guidance (age <5 years or having at least one underlying medical condition)^†††^ (*5*). Data were collected through parent or guardian interviews with assent from the child (when applicable) and medical chart reviews. Cases with missing influenza antiviral treatment data were excluded.

### Data Analysis

Influenza antiviral treatment was defined as documentation of prescription for or receipt of baloxavir, oseltamivir, peramivir, or zanamivir among persons in outpatient or inpatient hospital settings,^§§§^ respectively. Percentages of persons treated were calculated by dividing the number of persons treated with or prescribed antivirals by the number for whom receipt of antiviral treatment was recommended.^¶¶¶^ For historical context, the percentages of hospitalized children and adolescents with influenza who received antiviral treatment by age groups during October 1–April 30 from the 2017–18 through the 2022–23 seasons**** were calculated. For FluSurv-NET, unweighted counts and weighted percentages are presented to account for the complex survey design.

SAS software (version 9.4; SAS Institute) was used to conduct the analysis. FluSurv-NET and NVSN activities were reviewed by CDC, deemed not research, and were conducted consistent with applicable federal law and CDC policy.^††††^^,^^§§§§^

## Results

### Inpatient Influenza Antiviral Treatment Trends

During the 2017–18 season, the overall percentage of hospitalized patients aged <18 years with laboratory-confirmed influenza who were treated with antiviral medications was 70% in NVSN and 86% in FluSurv-NET (Figure). Since the 2019–20 season, the percentage of children and adolescents with influenza receiving treatment has declined and has remained lower than it was in seasons before the COVID-19 pandemic.

**FIGURE:**
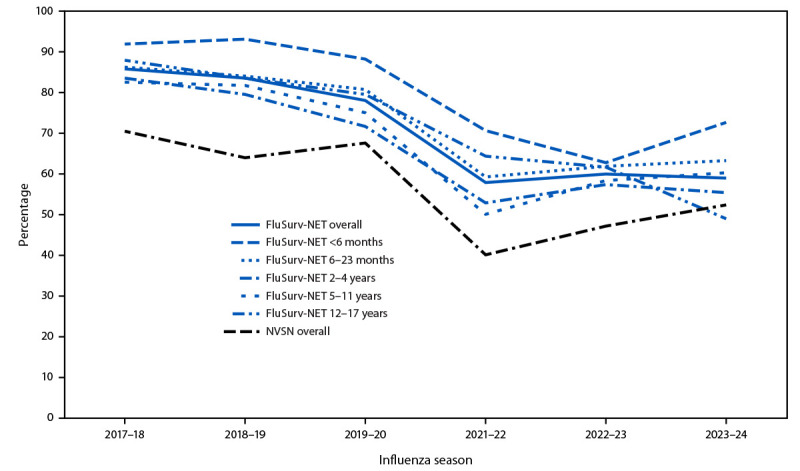
Antiviral treatment among children and adolescents aged <18 years hospitalized with laboratory-confirmed influenza, overall and by age group — two multistate surveillance networks,* United States, 2017–18 to 2023–24 influenza seasons^†^^,^^§^ **Abbreviations**: FluSurv-NET = Influenza Hospitalization Surveillance Network; NVSN = New Vaccine Surveillance Network. * Data presented overall for NVSN, and overall and by age groups for FluSurv-NET. Unless indicated, data included are from FluSurv-NET. ^†^ Clinical data were available on sampled FluSurv-NET cases for persons admitted during October 1–April 30 each season. This includes 8,907 children and adolescents in total, with 1,827 from 2017–18; 1,797 from 2018–19; 1,864 from 2019–20; 337 from 2021–22; 1,236 from 2022–23; and 1,846 from 2023–24. The 2020–21 influenza season was excluded because of minimal influenza activity. ^§^ Clinical influenza positive inpatient data were available from children and adolescents in NVSN admitted from October 1 through April 30 each season. This includes 1,175 children and adolescents in total, with 186 from 2017–18; 169 from 2018–19; 268 from 2019–20; 65 from 2021–22; 204 from 2022–23; and 283 from 2023–24. The 2020–21 influenza season was excluded because of minimal influenza activity.

### Characteristics of Children and Adolescents in FluSurv-NET and NVSN

During the 2023–24 influenza season, 573 influenza-associated outpatient visits and 283 influenza-associated hospitalizations in NVSN and 1,846 influenza-associated hospitalizations in FluSurv-NET were analyzed (Table 1).^¶¶¶¶^ Among children and adolescents with influenza-associated hospitalizations in NVSN and FluSurv-NET, the largest percentages of patients were aged 5–11 years (42% and 39%, respectively) and were non-Hispanic White persons (36% and 33%, respectively). In the outpatient setting, most children with influenza (42%) were aged 2–4 years and most (43%) were non-Hispanic Black or African American persons. Within NVSN and FluSurv-NET, 58% and 47% of hospitalized children and adolescents with influenza, respectively, did not have any underlying medical condition. Asthma or reactive airway disease was a frequently observed medical condition across networks and settings (21% in NVSN and 26% in FluSurv-NET). Among the hospitalized children and adolescents, 16% (NVSN) and 19% (FluSurv-NET) were admitted to an ICU, and 7%–13% (NVSN) and 4%–7% (FluSurv-NET) received invasive or noninvasive mechanical ventilation.

**TABLE 1:** Characteristics of children and adolescents with medically attended laboratory-confirmed influenza, by setting — two multistate surveillance networks, United States, 2023–24 influenza season

Characteristic	Total unweighted no. (col. %*)		
	NVSN		FluSurv-NET
	Outpatient^† ^n = 573	Inpatient n = 283	Inpatient N = 1,846
**Age group**			
<6 mos	37 (6)	22 (8)	175 (7)
6–23 mos	133 (23)	55 (19)	354 (17)
2–4 yrs	241 (42)	54 (19)	414 (22)
5–11 yrs	114 (20)	118 (42)	677 (39)
12–17 yrs	48 (8)	34 (12)	226 (15)
**Sex**			
Female	247 (43)	121 (43)	775 (44)
Male	326 (57)	162 (57)	1,071 (56)
**Race and ethnicity^§^**			
American Indian or Alaska Native	0 (—)	1 (0)	16 (1)
Asian or Pacific Islander	11 (2)	9 (3)	107 (5)
Black or African American	249 (43)	70 (25)	522 (31)
White	89 (16)	102 (36)	597 (33)
Hispanic or Latino	180 (31)	75(27)	498 (25)
Multiracial	32 (6)	20 (7)	24 (1)
Unknown	12 (2)	6 (2)	82 (4)
**ARI at admission** ^¶^			
Yes	—	—	1,694 (92)
No	—	—	152 (8)
**Underlying medical conditions**			
Asthma or reactive airway disease	122 (21)	61 (21)	428 (26)
Blood disorders	17 (3)	14 (5)	89 (5)
Cardiovascular disease	14 (2)	16 (6)	129 (7)
Chronic lung disease	128 (22)	71 (25)	98 (6)
Chronic metabolic disease	11 (2)	9 (3)	76 (4)
Immunocompromised	3 (1)	6 (2)	83 (5)
Neurologic disorders	33 (6)	33 (12)	319 (20)
Prematurity (aged <24 mos)	11 (3)	10 (13)	95 (19)
No. of underlying medical condition categories**			
0	370 (65)	164 (58)	947 (47)
1	176 (31)	84 (30)	620 (36)
2	20 (3)	23 (8)	164 (12)
≥3	7 (1)	12 (4)	85 (5)
**Hospital outcomes**			
ICU admission	—	44 (16)	362 (19)
ECMO	—	2 (5)	7 (0)
Noninvasive mechanical ventilation^††^	—	16 (13)	125 (7)
Invasive mechanical ventilation	—	9 (7)	67 (4)
In-hospital death	—	0 (—)	14 (1)

**Abbreviations:** ARI = acute respiratory illness; ECMO = extracorporeal membrane oxygenation; FluSurv-NET = Influenza Hospitalization Surveillance Network; ICU = intensive care unit; NVSN = New Vaccine Surveillance Network.

* Weighted percentages are calculated using sampling weights for FluSurv-NET.

^†^ Including outpatient clinics, urgent care clinics, and emergency departments. Only children and adolescents recommended to receive antiviral medication in the outpatient setting are included (specifically those aged <5 years or any child or adolescent with illness in one of the following medical condition categories: asthma or reactive airway disease, chronic lung disease, cancer, organ transplant, chronic metabolic disease, blood disorders, cardiovascular disease, neurologic disorders, immunocompromised, renal disease, liver disease, or obesity [among children aged ≥2 years]).

^§^ Persons of Hispanic or Latino (Hispanic) origin might be of any race but are categorized as Hispanic; all racial groups are non-Hispanic.

^¶^ For FluSurv-NET, ARI includes any of the following: fever, cough, nasal congestion, chest congestion, sore throat, hemoptysis, wheezing, apnea, cyanosis, difficulty breathing, nasal flaring, grunting, retractions, stridor, or shortness of breath. ARI at admission is part of the inclusion criteria for NVSN.

** Calculated as the number of conditions across categories including asthma or reactive airway disease, chronic lung disease, cancer, organ transplant, chronic metabolic disease, blood disorders, cardiovascular disease, neurologic disorders, immunocompromised, renal disease, liver disease, obesity (among children and adolescents aged ≥2 years), and prematurity (among those aged <24 months).

^††^ Bilevel positive airway pressure or continuous positive airway pressure.

### Influenza Antiviral Treatment During the 2023–24 Season

In the outpatient setting, 31% of children and adolescents who were recommended to receive antiviral treatment were prescribed antivirals (Table 2). The percentage of prescriptions was highest among children aged <6 months (49%) and lowest among those aged 2–4 years (21%); all outpatient prescriptions were for oseltamivir (Supplementary Table, https://stacks.cdc.gov/view/cdc/168887).

**TABLE 2:** Number and percentage of children and adolescents with medically attended, laboratory-confirmed influenza who received antiviral treatment, by setting — two multistate surveillance networks, United States, 2023–24 influenza season

Characteristic	Receipt of antiviral treatment, no./No. (%)*		
	NVSN		FluSurv-NET
	Outpatient^†^	Inpatient	Inpatient
**Overall**	**177/573 (31)**	**148/283 (52)**	**1,136/1,846 (59)**
**Age group**			
<6 mos	18/37 (49)	15/22 (68)	132/175 (73)
6–23 mos	51/133 (38)	26/55 (47)	223/354 (63)
2–4 yrs	51/241 (21)	23/54 (43)	244/414 (55)
5–11 yrs	39/114 (35)	64/118 (54)	401/677 (60)
12–17 yrs	19/48 (40)	20/34 (59)	136/226 (49)
**Sex**			
Female	66/247 (27)	64/121 (53)	484/775 (59)
Male	111/326 (33)	84/162 (52)	352/1,071 (60)
**Race and ethnicity** ^§^			
American Indian or Alaska Native	0 (—)	1/1 (100)	9/16 (53)
Asian or Pacific Islander	6/11 (54)	2/9 (22)	64/107 (58)
Black or African American	61/249 (25)	31/70 (44)	326/522 (59)
White	34/89 (38)	59/102 (58)	354/597 (54)
Hispanic or Latino	69/180 (38)	43/75 (57)	320/498 (66)
Multiracial	6/32 (19)	11/20 (55)	16/24 (50)
Unknown	1/12 (8)	1/6 (17)	47/82 (84)
**High-risk category**			
Aged <5 years	101/362 (28)	27/65 (42)	351/584 (57)
High-risk condition^¶^	57/162 (35)	43/92 (47)	358/540 (61)
Aged <5 years and high-risk condition	19/79 (39)	57/87 (65)	248/359 (66)
No additional high-risk factors	—	21/39 (54)	179/363 (51)
**Underlying medical conditions**			
Asthma or reactive airway disease	41/122 (34)	39/61 (64)	280/428 (62)
Blood disorders	5/17 (29)	7/14 (50)	74/89 (83)
Cardiovascular disease	7/14 (50)	13/16 (81)	102/129 (75)
Chronic lung disease	45/128 (35)	46/71 (65)	82/98 (75)
Chronic metabolic disease	8/11 (73)	5/9 (56)	54/76 (62)
Immunocompromised	2/3 (67)	3/6 (50)	61/83 (72)
Neurologic disorders	13/33 (39)	21/33 (64)	208/319 (59)
Prematurity (aged <24 mos)	5/11 (45)	4/10 (40)	73/95 (74)
No. of underlying medical conditions**			
0	104/370 (28)	73/164 (45)	530/947 (55)
1	59/176 (34)	51/84 (61)	394/620 (60)
2	10/20 (50)	15/23 (65)	144/194 (66)
≥3	4/7 (57)	9/12 (75)	68/85 (77)
**Hospital outcomes**			
ICU admission	—	37/44 (84)	301/362 (82)
ECMO	—	2/2 (100)	6/7 (86)
Noninvasive mechanical ventilation^††^	—	15/16 (94)	115/125 (82)
Invasive mechanical ventilation	—	8/9 (89)	58/67 (82)
In-hospital death	—	0 (—)	12/14 (86)

**Abbreviations:** ECMO = extracorporeal membrane oxygenation; FluSurv-NET = Influenza Hospitalization Surveillance Network; ICU = intensive care unit; NVSN = New Vaccine Surveillance Network.

* Weighted percentages calculated using sampling weights for FluSurv-NET.

^†^ Including outpatient clinics, urgent care clinics, and emergency departments. Only children and adolescents recommended to receive antiviral medication in the outpatient setting are included (specifically those aged <5 years or any child or adolescent with an illness in one of the following medical condition categories: asthma or reactive airway disease, chronic lung disease, cancer, organ transplant, chronic metabolic disease, blood disorders, cardiovascular disease, neurologic disorders, immunocompromised, renal disease, liver disease, or obesity [among children and adolescents aged ≥2 years]).

^§^ Persons of Hispanic or Latino (Hispanic) origin might be of any race but are categorized as Hispanic; all racial groups are non-Hispanic.

^¶^ Includes the following medical condition categories: asthma or reactive airway disease, chronic lung disease, cancer, organ transplant, chronic metabolic disease, blood disorders, cardiovascular disease, neurologic disorders, immunocompromised, renal disease, liver disease, obesity (among children aged ≥2 years), and prematurity (among those aged <24 months).

** Calculated as the number of conditions across categories including asthma or reactive airway disease, chronic lung disease, cancer, organ transplant, chronic metabolic disease, blood disorders, cardiovascular disease, neurologic disorders, immunocompromised, renal disease, liver disease, obesity (among children and adolescents aged ≥2 years), and prematurity (among those aged <24 months).

^††^ Bilevel positive airway pressure or continuous positive airway pressure.

Among children and adolescents hospitalized with influenza, 52% (NVSN) and 59% (FluSurv-NET) received antiviral treatment (Table 2). Antiviral treatment prevalence among hospitalized children and adolescents with influenza was highest among those aged <6 months (68% [NVSN]; 73% [FluSurv-NET]) and lowest among those aged 2–4 years in NVSN (43%) and 12–17 years in FluSurv-NET (49%). Nearly all treated patients received oseltamivir (99%) (Supplementary Table, https://stacks.cdc.gov/view/cdc/168887); most (68% [NVSN]; 60% [FluSurv-NET]) received treatment on the day of admission, and 29% (NVSN) and 34% (FluSurv-NET) were not treated until ≥1 day after admission.

In both outpatient and inpatient settings, the percentage of children and adolescents who received antiviral treatment for laboratory-confirmed influenza rose with an increasing number of underlying medical conditions, from 28% of those with no underlying conditions to 57% among those with three or more (outpatient) and, among hospitalized patients, from 45% to 75% (NVSN), respectively, and from 55% to 77% (FluSurv-NET), respectively (Table 2). The percentage of patients with underlying medical conditions who received antiviral treatment varied by condition, network, and setting. Among those with asthma or reactive airway disease (a frequent comorbidity between networks and settings), 34% of those in outpatient settings were prescribed antivirals, and 64% (NVSN) and 62% (FluSurv-NET) of those who were hospitalized received antiviral treatment.

In children and adolescents who were hospitalized, a higher proportion of those admitted to an ICU received antiviral treatment (84% [NVSN]; 82% [FluSurv-NET]); 81% of those admitted were treated on the day of or after ICU admission. Among those who received noninvasive or invasive ventilation, 89%–94% in NVSN and 82% in FluSurv-NET received antiviral treatment.

## Discussion

Seasonal influenza causes substantial disease among children and adolescents in the United States each year, and annual influenza vaccination is recommended for all persons aged ≥6 months, including those who are pregnant (to protect themselves and their infants aged <6 months through passive transplacentally transferred antibodies) (*7**)*. Antiviral treatment is an important adjunct to reduce the risk for influenza complications. Among patients with confirmed or suspected influenza, initiation of antiviral treatment is recommended as soon as possible for outpatients at higher risk for influenza complications and for all hospitalized patients (*4*,*5*). The percentage of children and adolescents with influenza-associated hospitalization who received antiviral treatment remained relatively stable from the 2017–18 season to the 2019–20 season, and subsequently decreased sharply during the COVID-19 pandemic. Although antiviral treatment has stabilized during the past two seasons, the percentages of patients treated have remained suboptimal and have not returned to prepandemic levels. During the 2023–24 influenza season, approximately one half (41%–48%) of children and adolescents with an influenza-associated hospitalization and approximately two thirds (69%) of those with an influenza-associated outpatient visit did not receive recommended antiviral treatment, highlighting missed opportunities to reduce the risk for influenza complications. This decrease in use of influenza antiviral treatment underscores the importance of increasing awareness among pediatric health care professionals about current recommendations for antiviral treatment.

Antiviral treatment is associated with improved outcomes for children and adolescents with influenza, including in-hospital survival (*2*,*3**,**8*). Antiviral treatment initiation shortly after influenza symptom onset provides more clinical benefit than does later treatment initiation (*2*,*3*). Among children and adolescents who are hospitalized or who are at higher risk for influenza-associated complications, there is no restriction on the timing of initiation of antiviral treatment, although starting as early as possible is recommended. Despite these recommendations, concerns about the timing of antiviral treatment relative to symptom onset and waiting for influenza test results have been noted as reasons for not prescribing antivirals among infants in the outpatient setting (*9*). Understanding of the reasons for nontreatment among hospitalized children and adolescents with influenza is limited, but some reasons might include concerns about adverse events (*10*). Increasing access to timely care, identifying potential barriers to antiviral treatment in the hospital setting, and increasing provider education concerning the benefits of timely treatment might lead to increases in antiviral treatment of persons who are recommended to receive it.

### Limitations

The findings in this report are subject to at least three limitations. First, FluSurv-NET and NVSN catchment areas do not cover the entire U.S. population; characteristics of children and adolescents with medically attended and laboratory-confirmed influenza infection might not be generalizable throughout the United States. Second, antiviral treatment before hospitalization might be recorded incompletely because patients might have received treatment in the outpatient setting. Finally, the calculation of antiviral treatment timing might be imprecise because only dates and not time of admission and treatment initiation were collected.

### Implications for Public Health Practice

Annual influenza vaccination provides important protection against influenza and associated complications. Among patients with confirmed or suspected influenza who are at higher risk for complications, early initiation of antiviral treatment is recommended to further reduce the risk for complications. The decrease in influenza antiviral use among children and adolescents with laboratory-confirmed influenza since the COVID-19 pandemic is concerning. Health care providers are reminded that children and adolescents with suspected or confirmed influenza who are hospitalized or have higher risk for influenza complications should receive prompt antiviral treatment.

## Footnotes

* These senior authors contributed equally to this report.

†
https://www.cdc.gov/flu-burden/php/data-vis/index.html

§
https://gis.cdc.gov/grasp/FluView/FluHospRates.html

¶ https://www.cdc.gov/fluview/overview/influenza-hospitalization-surveillance.html; https://www.cdc.gov/nvsn/php/about/index.html

** Defined as receipt of a positive test result from a viral culture, direct or indirect fluorescent antibody staining, rapid antigen test, molecular assay, or evidence of a positive influenza test result in a clinical note within 14 days before or during hospitalization.

†† Patients in FluSurv-NET lived in catchment area counties from the following states: California, Colorado, Connecticut, Georgia, Maryland, Michigan, Minnesota, New Mexico, New York, Oregon, Tennessee, and Utah.

§§ Vanderbilt University Medical Center, Nashville, Tennessee; University of Rochester School of Medicine and Dentistry-Medical Center/UR-Golisano Children’s Hospital, Rochester, New York; Cincinnati Children's Hospital Medical Center, Cincinnati, Ohio; Texas Children's Hospital, Houston, Texas; Seattle Children's Hospital, Seattle, Washington; Children's Mercy Hospital, Kansas City, Missouri; Children's Hospital of Pittsburgh, Pittsburgh, Pennsylvania.

¶¶ ARI is defined as at least one of the following within 10 days of the medical encounter: fever, cough, earache, nasal congestion, runny nose, sore throat, wheezing, shortness of breath or rapid shallow breathing, apnea, apparent life-threatening event, or briefly resolved unexplained event.

*** Defined as receipt of a positive test result from a rapid antigen or polymerase chain reaction test or a molecular assay.

††† Asthma or reactive airway disease, chronic lung disease, cancer, organ transplant, chronic metabolic disease, blood disorders, cardiovascular disease, neurologic disorders, immunocompromised, renal disease, liver disease, or obesity among children and adolescents aged ≥2 years).

§§§ For FluSurv-NET, this could be up to 2 weeks before or at any time during hospitalization.

¶¶¶ Includes all persons with influenza who were hospitalized or, in the outpatient setting, those aged <5 years, or with illness in one of the following medical condition categories: asthma or reactive airway disease, chronic lung disease, cancer, organ transplant, chronic metabolic disease, blood disorders, cardiovascular disease, neurologic disorders, immunocompromised, renal disease, liver disease, or obesity (among children aged ≥2 years).

**** The 2020–21 influenza season was excluded because of minimal influenza activity.

†††† 45 C.F.R. part 46.102(l)(2), 21 C.F.R. part 56; 42 U.S.C. Sect. 241(d); 5 U.S.C. Sect. 552a; 44 U.S.C. Sect. 3501 et seq.

§§§§ FluSurv-NET and NVSN sites obtained human subjects and ethics approval from their respective state health departments, academic partners, or participating hospital institutional review boards.

¶¶¶¶ In FluSurv-NET, a total of 1,026 cases were excluded: 954 were not sampled, 14 sampled cases were missing antiviral use data, 50 sampled cases were considered nosocomial (positive influenza test result >3 days after hospital admission), and eight persons with sampled cases were pregnant.
